# Latent profile analysis of college students’ healthy lifestyles and its association with physical activity

**DOI:** 10.1186/s12889-025-24733-6

**Published:** 2025-10-07

**Authors:** Dongyue Wang, Rui Ma, Yafei Li, Yani Li

**Affiliations:** 1 Sports Department of Zhengzhou University of Aeronautics, Zhengzhou University, Zhengzhou, Henan Province 450001 China; 2 School of Physical Education and Sports, Zhengzhou University, Zhengzhou, Henan Province 450015 China; 3 School of Economics, Shen Zhen MSU-BIT University, Shen Zhen City, Guangdong Province China; 4 School of Business, East China University of Science and Technology, Shang Hai City, China

**Keywords:** Healthy lifestyle, Physical activity, Latent profile analysis, Regression analysis, Freshman students

## Abstract

**Objective:**

This study investigates the latent profiles of healthy lifestyles among college students and examines their associations with physical activity levels, thereby establishing a scientific foundation for developing targeted interventions to address sub-health conditions.

**Methods:**

From October to December 2024, a stratified random cluster sampling method was used to select 2,058 college students from two representative universities in Henan Province. Data were collected through validated questionnaires, including the College Students’ Healthy Lifestyle Assessment Scale (CSHLAS) and the short-form International Physical Activity Questionnaire (IPAQ-SF). Data analysis was performed using Mplus 8.3 and SPSS 26.0 software.

**Results:**

College students’ healthy lifestyles were classified into three latent profiles: Health Crisis Type (15.65%), Sub-health Adjustment Type (56.26%), and Healthy Vitality Type (28.09%). Statistically significant differences in the distribution of lifestyle profiles were observed across students’ majors, family living areas, and parental education levels (all *P* < 0.01). Compared with the Healthy Vitality Type, the risk of low physical activity was 6.99 times (95% CI = 3.84–12.71) and 2.29 times (95% CI = 1.59–3.28) higher in the Health Crisis Type and Sub-health Adjustment Type, respectively (both *P* < 0.001). The risk of moderate physical activity was 2.66 times (95% CI = 1.52–4.66) and 1.54 times (95% CI = 1.14–2.08) higher, respectively (both *P* < 0.01).

**Conclusions:**

Insufficient physicalactivity among college students is a significant issue, and their healthy lifestyle behaviors exhibit heterogeneity, which is significantly associated with physical activity levels. Targeted, individualized, and precise interventions should be developed, particularly for students from rural areas and those majoring in the humanities and medical sciences, in order to reduce the adoption of unhealthy lifestyles.

## Introduction

A healthy lifestyle is a core determinant of lifelong health, with its cultivation being especially critical during early adulthood [1]. From the perspective of the life course theory, the behavioral patterns formed during this period have a profound impact on an individual’s long-term health trajectory. Pender defined it as a set of actions individuals take to achieve positive health outcomes [2]; the World Health Organization (WHO) emphasizes that it is a lifelong behavior pattern formed through the dynamic interaction of complex sociocultural contexts, personal characteristics, and living conditions [3]. In line with current consensus, a healthy lifestyle is generally characterized by a series of beneficial and interconnected habitual behaviors that promote both physical and mental health. The core dimensions typically encompass adequate physical activity, balanced nutrition, quality sleep, avoidance of tobacco, limitation of alcohol consumption, effective stress management, positive social relationships, and active health responsibility [4, 5].

The university stage is considereda critical window for shaping healthy lifestyles and preventing future Non-Communicable Diseases (NCDs) [6]. NCDs, including cardiovascular diseases and metabolic disorders, diabetes, and chronic respiratory diseases, collectively account for 71% of global mortality and 46% of morbidity [7]. However, this period is frequently marked by the adoption and persistence of unhealthy lifestyles. Extensive evidence indicates that college students commonly exhibit physical inactivity (PI), poor sleep quality, poor dietary habits, and tobacco or alcohol consumption. These behaviors adversely impact academic performance and mental health while elevating future NCD risk, thereby contributing to a pressing global public health burden [8].

The health status of Chinese college students is equally concerning. According to the 2019 China Student Physical Fitness and Health Survey, college students’ physical fitness has steadily declined since 1985, accompanied by rising rates of overweight and obesity [9]. Numerous studies identify physical inactivity (PI) as a key driver of these issues, contributing to reduced physical fitness, elevated obesity rates, and heightened risks of anxiety and depression [10–13]. Notably, physical inactivity is an established risk factor for global mortality [14]. Globally, about 25% of adults do not meet the recommended physical activity levels by the WHO, and the issue of physical inactivity is even more severe in the college student population. For example, at the University of California, 84.8% of students’ waking time is spent in sedentary behavior [15]; a study covering nearly 18,000 college students from 23 countries revealed an overall physical inactivity (PI) rate of 41.4%, with Pakistan reaching as high as 80.6% [16]. In the UK, 73% of Male and 79% of female students do not meet the physical activity guidelines [17], while 28.5% of nursing students in Germany engage in less than one physical activity session per week [18]. In China, a nationwide survey covering 25,000 college students in 2023 showed that nearly 50% of students exercised less than three times per week [19]. More critically, modern lifestyles have triggered a ‘fragile’ phenomenon among Chinese college students [20], characterized by physical vulnerability, prevalent anxiety and depression, and high obesity rates, with PI being a central exacerbating factor [21, 22]. Thus, interventions to enhance health literacy and motivate lifestyle modifications in college students must be prioritized in public health agendas.

Despite the growing body of research on college students’ healthy lifestyles, significant disagreements persist in the conclusions drawn from existing studies. One key limitation is the prevalent use of variable-centered approaches. These methods primarily focus on analyzing linear associations between individual health behaviors (e.g., physical activity, PA) and other variables (e.g., BMI, mental health) or exploring correlations among multiple variables. However, variable-centered approaches fail to effectively uncover potential heterogeneous subgroups based on multidimensional health behavior patterns within the population. This oversight of heterogeneity severely limits our understanding of the specific characteristics, formation mechanisms, and key drivers of healthy lifestyles in different subgroups of college students. In particular, physical activity (PA)—a core dimension of healthy lifestyles—plays a significant role in various behavior pattern combinations, interacts with other health behaviors (e.g., sleep, stress management), and contributes to the differentiation of healthy lifestyle subgroups. However, traditional research methods struggle to reveal these dynamics.

Given the high prevalence of physical inactivity (PI) among college students, its strong co-occurrence with other health risk behaviors (e.g., poor diet, sleep deprivation), and significant shortcomings of existing variable-centered methods in capturing heterogeneity in group behavior patterns, this study proposes the following core questions:

What latent categories of healthy lifestyles exist among Chinese college students, characterized by significant differences in their manifestations across multiple core dimensions?

What are the specific associations between these different healthy lifestyle categories and physical activity levels?

To scientifically address the above questions, this study innovatively adopts an individual-centered research perspective and applies Latent Profile Analysis (LPA), an advanced statistical modeling technique. LPA can identify latent subgroups within a population based on individuals’ performance patterns across multiple core dimensions of healthy lifestyles, thereby effectively revealing the heterogeneity of complex group structures based on behavior combinations with higher internal homogeneity [23]. This study aims to go beyond the isolated examination of single behaviors and provide an in-depth analysis of the complexity of healthy lifestyle behaviors among Chinese college students. The expected findings will provide a solid scientific basis for universities to develop precise, differentiated health promotion intervention strategies, thereby more effectively supporting students with different needs in adopting and maintaining healthy habits, ultimately improving overall health levels and future well-being.

To scientifically address the above questions, this study innovatively adopts an individual-centered research perspective and applies Latent Profile Analysis (LPA), an advanced statistical modeling technique. LPA can identify latent subgroups within a population based on individuals’ behavioral patterns across multiple core dimensions of healthy lifestyles, thereby effectively revealing the heterogeneity among complex group structures—where each subgroup is characterized by behavior combinations with higher internal homogeneity [24]. This study aims to go beyond the isolated examination of single behaviors and provide an in-depth analysis of the complexity of healthy lifestyle behaviors among Chinese college students. The expected findings will provide a solid scientific basis for universities to develop precise and differentiated health promotion intervention strategies, thereby more effectively supporting students with different needs in adopting and maintaining healthy habits, and ultimately improving their overall health levels and future well-being.

## Study subjects and methods

### Study subjects

This study was conducted from October to December 2024 at two universities classified as “Double First Class” in Henan Province, China. The purpose was to investigate the health lifestyles and physical activity of first-year students. Considering the nature of the universities (comprehensive universities), the representativeness of academic disciplines (liberal arts, science, engineering, and medicine), and the feasibility of sampling, this study employed a stratified random cluster sampling method.

### The specific steps of the sampling method were as follows

The first-year student classes of the two universities were categorized into four strata based on academic disciplines: liberal arts, science, engineering, and medicine.

Within each discipline, administrative classes were defined as sampling clusters. The research team obtained the class rosters of all eligible first-year classes from the academic affairs offices of the two universities and categorized them by discipline. All classes within each stratum were numbered, and seven classes were randomly selected from each discipline using a lottery method. This process ensured that each class had an equal chance of being selected within its stratum. A total of 56 classes, with 2,136 students, were surveyed. Based on preset standards, data with incomplete information, unusually short response times, or patterns of consistent answers were excluded through strict screening, resulting in the final collection of 2,058 valid questionnaires, with an effective response rate of 92.04%. This study was approved by the Biomedical Research Ethics Committee of Zhengzhou University (Approval No.: 2024 − 148). Before data collection, all participating students were fully informed of the research purpose and procedures and signed a written informed consent form.

## Survey instruments

### General information survey

Based on a review of the literature on the demographic variables influencing healthy lifestyles [25, 26] and considering the actual situation, the general information survey was designed. It mainly includes the following variables: gender, major, place of origin, whether the student is an only child, and parental education level.

### College students’ healthy lifestyle assessment scale

The College Students’ Healthy Lifestyle Assessment Scale used in this study was developed and validated by Jiao Jianpeng and Wang Dongli through three rounds of expert interviews [27]. The scale consists of 33 items, covering 8 core dimensions: exercise behavior, regularity of daily routines, nutritional intake, health risk behaviors, health Management awareness, interpersonal communication, stress regulation ability, and life values. Among the 33 items, 31 are positive items and 2 are reverse-scored items. A 5-point Likert scale was used for scoring, where 1–5 represent “never,” “occasionally,” “about half the time,” “often,” and “always,” respectively. The total score range of the scale is 33 to 165, with higher scores indicating healthier lifestyles. The Cronbach’s α coefficient of the scale is 0.898, and the split-half reliability coefficient is 0.840. This scale provides a reliable tool for assessing multiple aspects of college students’ healthy lifestyles and serves as a robust measurement tool for related research.

### International physical activity questionnaire (IPAQ)

This study used the revised version of the International Physical Activity Questionnaire (IPAQ) to measure the physical activity levels of the survey participants [28]. The short form of the IPAQ, which is a self-reported questionnaire, is certified by the WHO Global Physical Activity Measurement System. It includes three modules: ①Activity Frequency-Duration Matrix (6 items): This module recalls physical activity in the past 7 days and classifies it into low, moderate, and vigorous physical activity. ② Metabolic Equivalent (MET) Conversion System: This module follows the American College of Sports Medicine (ACSM) standardized energy expenditure guidelines, setting MET values for walking (3.3 METs), moderate intensity (4.0 METs), and vigorous intensity (8.0 METs).③ Energy Expenditure Calculation System: Using the MET-min/week calculation process certified by ISO 20712:2019, the specific formula for total energy expenditure is: Total energy expenditure (MET-min/week) = Σ (duration of each activity × weekly frequency × corresponding MET value).

### Latent profile analysis

Latent Profile Analysis (LPA) is an individual-centered modeling approach that identifies latent subgroups based on an individual’s responses to observed variables. It uses objective statistical fit indices to assess the accuracy and validity of the classifications, aiming to maximize heterogeneity between groups and homogeneity within groups. Commonly used model fit indices include: Akaike Information Criterion (AIC), Bayesian Information Criterion (BIC), adjusted Bayesian Information Criterion (aBIC), Lo-Mendell-Rubin likelihood ratio test (LMRT), Bootstrapped Likelihood Ratio Test (BLRT), and entropy. Among these, AIC, BIC, and aBIC are indicators of model fit quality; smaller values indicate better model fit. Entropy reflects classification quality, with values closer to 1 indicating more accurate classification [29]. LMRT and BLRT are used to compare models with M classes and M-1 classes; a P-value greater than 0.05 suggests a better fit for the M-class model. When the optimal models suggested by various indices are inconsistent, a comprehensive judgment is required [30].

## Data collection and quality control

Before the survey, 35 physical education assistant teachers received standardized training, followed by a pre-survey. Based on the feedback from the pre-survey data, the research team developed and refined the standardized assessment tool through three rounds of revision and validation. A multi-level communication mechanism was established between the project leader and the Department of Public Physical Education. First, targeted training was conducted on the research purpose, target population, and implementation standards. Second, a quality control team was formed. Finally, after the approval of the teaching team, the data collection procedure was implemented synchronously online and offline. Each device and IP address was limited to a single response. Researchers monitored the data in real time and conducted a double-entry verification process to ensure data accuracy and integrity.

### Statistical analysis

Data cleaning and statistical analysis were conducted using SPSS 26.0, and Latent Profile Analysis (LPA) was performed using Mplus 8.3. Descriptive statistics, independent sample t-tests, and one-way ANOVA were used to compare differences in demographic variables. A robust three-step method (R3STEP) was used to conduct multinomial logistic regression analysis on demographic variables [31]. Multiple linear regression analysis was employed to examine the association between college students’ healthy lifestyle categories and physical activity and its dimensions. All models underwent multicollinearity diagnostics and model fit testing (VIF < 2), with a significance level set at *P* < 0.05.

## Results

### Sample Characteristics

Among the 2,058 participants, 1,087 were male (52.8%) and 971 were female (47.2%). A total of 1,003 students were from urban areas (48.7%), and 1,055 were from rural areas (51.3%). In terms of academic Majors, 656 students were in the humanities (31.9%), 495 in the natural sciences (24.1%), 746 in engineering (36.2%), and 161 in medicine (7.8%). Regarding family structure, 410 students were only children (19.9%), and 1,648 were non-only children (80.1%). As for parental education levels, 343 students (16.7%) had parents with a bachelor’s degree or higher, 237 students (11.5%) had parents with a college diploma, 541 students (26.3%) had parents with a high school education, and 937 students (45.5%) had parents with a junior high school education or lower (see Table 1).

**Table 1:** Characteristics of the study participants

Variable		Number(*N*)	Percentage(%)
**Gender**	Male	1087	52.8
	Female	971	47.2
**Place of origin**	Urban	1003	48.7
	Rural	1055	51.3
**Academic major**	Humanities	656	31.9
	Natural sciences	495	24.1
	Engineering	746	36.2
	Medical sciences	161	7.8
**Only child status**	Yes	410	19.9
	No	1648	80.1
**Parental education**	Bachelor or above	343	16.7
	Associate degree	237	11.5
	High school	541	26.3
	Junior high or below	937	45.5

### Analysis of college students’ healthy lifestyle and physical activity status

The overall mean score for the healthy lifestyle of 2,058 college students was (3.48 ± 0.58). The average scores for each dimension were as follows: health responsibility behaviors (3.85 ± 0.72), interpersonal relationship behaviors (3.80 ± 0.81), stress management behaviors (3.71 ± 0.78), life appreciation behaviors (3.66 ± 0.89), dietary nutrition behaviors (3.23 ± 0.86), regular lifestyle behaviors (3.18 ± 0.93), health risk behaviors (3.06 ± 0.32), and exercise behaviors (2.52 ± 0.88). The average physical activity level of college students was at a moderate-to-low level (mean intensity: 1.87 ± 0.56). Specifically, 65.3% of students reported a moderate level of physical activity, 23.9% reported a low level, and only 10.9% engaged in high physical activity.

### Latent profile analysis of college students’ healthy lifestyle

Based on the multidimensional healthy lifestyle assessment scale, latent profile analysis was performed using a systematic process and model fit diagnostics. The fit indices are shown in Table 2. From Model 1 to Model 4, the fit indices showed a typical pattern of change: as the number of latent categories increased, the information criteria (AIC = 81836.253→76240.288, BIC = 81926.325→76482.356, aBIC = 81875.492→76345.741) decreased, while the entropy value (0.80→0.84) continuously improved, reaching the threshold for discriminant validity (> 0.8). Moreover, the likelihood ratio tests (LMR < 0.001, BLRT < 0.001) were statistically significant. However, Model 4 showed an imbalance in subgroup structure (the smallest category accounted for 4.81%, which is below the 5% threshold for regional latent category analysis), indicating that this model did not have meaningful classification. Therefore, a three-class model (Model 3) was determined to be the best fit.

**Fig. 1:**
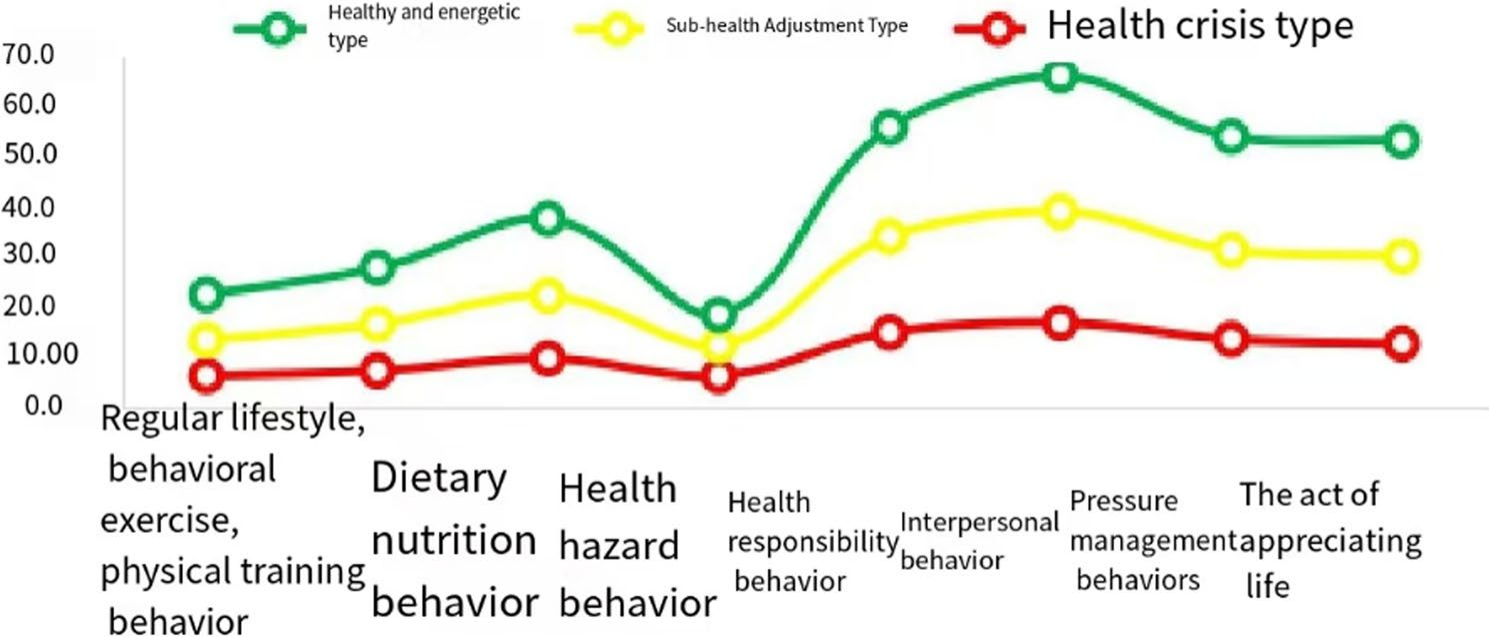
distribution of characteristics of 3 potential categories of healthy lifestyles among college students

A latent profile visualization analysis based on the classification results is shown in Fig. 1. The categories were named according to the average scores of each dimension. The C1 category, consisting of 322 students (15.65%), exhibited the lowest and below-average scores across the standardized behavior dimensions; thus, it was named the “Health Crisis Type.” The C2 category, consisting of 1,158 students (56.26%), was the largest group, with average scores at the moderate level across dimensions, but exhibiting behaviors such as low physical activity, irregular routines, inadequate nutrition, low responsibility, light management, weak interpersonal relationships, and minimal life appreciation. Therefore, it was named the “Sub-health Adjustment Type.” The C3 category, consisting of 578 students (28.09%), exhibited the highest scores across all dimensions, surpassing the average, and was therefore named the “Healthy Vitality Type.”

**Table 2:** Indicators of potential profile model fit for healthy lifestyles of college students

model	AIC	BIC	aBIC	Entropy	LMRT	BLRT	Category probability
1	81836.253	81926.325	81875.492	—	—	—	100
2	78363.054	78503.791	78424.364	0.80	< 0.001	< 0.001	50.34/49.66
3	76959.959	77151.362	77043.341	0.84	< 0.001	< 0.001	15.65/28.08/56.27
4	76240.288	76482.356	76345.741	0.84	< 0.001	< 0.001	4.71/43.44/30.71/21.14

### Analysis of differences in the distribution of potential healthy lifestyle categories among college students

The results showed that the “Adjustment Type” was the most common healthy lifestyle state among college students. Engineering students had the highest proportion of the “Crisis Type” (16.6%), while medical students had the lowest proportion of the “Vitality Type” (23.6%). College students from rural areas had the highest proportion of the “Crisis Type” (17.5%) and the lowest proportion of the “Vitality Type” (24.1%). College students with lower parental education levels (especially those with parents having only junior high school education or lower) were also more likely to face challenges with a higher proportion of the “Crisis Type” and a lower proportion of the “Vitality Type.” Non-only children had a significantly lower proportion of the “Vitality Type” (26.0%) compared to only children (36.3%). The results of the Chi-square test showed that the differences in the distribution of potential healthy lifestyle categories among college students with different academic majors, places of origin, and parental education levels were statistically significant (P values all < 0.01). See Table 3.

**Table 3:** Comparison of the distribution of potential healthy lifestyle categories among college students with different demographic characteristics

Project		Overall	Crisis type	Adaptability	The dynamic type	χ༒ value	Pvalue
gender	male	1087	163(15.0%)	603(55.5%)	321(29.5%)	2.59	0.273
	Female	971	159(16.4%)	555(57.2%)	257(26.5%)		
major	Science	495	80(16.2%)	273(55.2%)	142(28.7%)	21.98	*P* < 0.01
	liberal arts	656	95(14.5%)	388(59.1%)	173(26.4%)		
	Engineering	746	124(16.6%)	397(53.2%)	225(30.2%)		
	Medical science	161	23(14.3%)	100(62.1%)	38(23.6%)		
Place of birth	Town	1003	137(13.7%)	542(54.0%)	324(32.3%)	19.06	*P* < 0.01
	Rural areas	1055	185(17.5%)	616(58.4%)	254(24.1%)		
Whether only child	Yes	410	52(12.7%)	209(51.0%)	149(36.3%)	5.33	0.069
	No	1648	270(16.4%)	949(57.6%)	429(26.0%)		
Parental educational level	Bachelor’s degree or above	343	43(12.5%)	173(50.4%)	127(37.0%)	21.99	*P* < 0.01
	junior college	237	35(14.8)	136(57.4%)	66(27.8%)		
	High school	541	75(13.9%)	318(58.8%)	148(27.4%)		
	Junior high school and below	937	169(18.0%)	531(56.7%)	237(25.3%)		

Note: ()The numbers inside represent the composition ratio %

### Association between college students’ healthy lifestyle categories and physical activity

Using physical activity intensity as the dependent variable (with high physical activity as the reference group), and the latent healthy lifestyle categories (with the “Vitality Type” as the reference group) as independent variables, a multivariate logistic regression model was employed, controlling for demographic factors such as gender, academic major, and place of origin. The results showed that, compared to students with a “Vitality Type” lifestyle, those with a “Crisis Type” or “Adjustment Type” lifestyle had 6.99 times and 2.29 times the risk, respectively, of being at a low level of physical activity, with these differences being highly significant (*P* < 0.001). The risk of being at a moderate level of physical activity was 2.66 times and 1.54 times higher, respectively, compared to “Vitality Type” students, with these differences also being very significant (*P* < 0.01). See Table 4.

Table 4 Multivariate Logistic Regression Analysis of the Association Between College Students’ Healthy Lifestyle Categories and Physical Activity.

**Table 4:** Multiple logistic regression analysis between different categories of healthy lifestyle and physical activity among college students

Independent variable	Low physical activity level	Moderate physical activity level
Health crisis type	6.99(3.84–12.71)***	2.66(1.52–4.66)**
Sub- health adjustment type	2.29(1.59–3.28)***	1.54(1.14–2.08)**

Note : ** *P* < 0.01, *** *P* < 0.001

## Discussion

This study identified a three-category structure of college students’ healthy lifestyles through Latent Profile Analysis (LPA): Health Crisis Type (15.65%), Sub-health Adjustment Type (56.26%), and Health Vitality Type (28.09%), with significant differences between categories. This indicates the heterogeneity of healthy lifestyles in this group. The “Adjustment Type” was revealed as a key transitional state, characterized by insufficient physical activity, irregular sleep patterns, unbalanced nutrition, weak health awareness, poor health management abilities, low frequency of social interactions, and insufficient recognition and appreciation of a healthy lifestyle. This finding is consistent with existing research [32], suggesting that most college students’ healthy lifestyles are at a moderate level, presenting an overall sub-healthy state.

This phenomenon may be closely related to the transformation of modern lifestyles, especially the widespread use of the internet and electronic products, increased sedentary behavior, and irregular daily routines among college students. It is worth noting that the study found that both the Vitality and Crisis Types have increased in proportion. This change could be related to various recent social policies and environmental factors. On the one hand, outbreaks of COVID-19 and other viral diseases [33], the frequent occurrence of the “fragile” phenomenon, and various competitive pressures [34] have led some college students to become more health-conscious, actively participating in fitness, mental health activities, and social practices, thus promoting the formation of healthy lifestyles. On the other hand, the influence of electronic lifestyles in the era of new media may lead to irregular routines, overweight and obesity, anxiety, depression, and other risks [35], exacerbating the formation of unhealthy lifestyles. Furthermore, the implementation of the strategies for building a strong sports nation, nationwide fitness, overall health, and healthy campuses has, to some extent, stimulated college students’ health awareness. However, differences in research subjects, variables, regions, and environments may also affect the results. Therefore, future studies should further explore how these factors influence college students’ healthy lifestyles and provide scientific evidence for the development of targeted health promotion strategies.

The study found that the “Adjustment Type” is the most common healthy lifestyle state among college students. The Vitality Type was most prevalent among STEM students, and significant differences were observed in the distribution of the “Crisis Type” and “Vitality Type” across different groups (major, place of origin, parental education level). This suggests that academic background, place of origin, and parental education level are important factors influencing college students’ healthy lifestyles, which aligns with related studies [36]. The empirical health view shaped by their academic culture may be a reason. STEM students tend to transfer their systematic problem-solving mindset to health management [37], using analytical cognition to enhance behavioral execution [38]. However, attention should be given to the development of their social-emotional skills [39, 40]. Compared to students from rural areas, urban students typically have access to more abundant health resources (such as national fitness facilities, mental health education, etc.) [41], and their relatively better financial status can directly translate into support for healthy food, sports equipment, and preventive medical services, thereby reducing economic barriers to healthy behaviors [42]. High-education families often embed health advantages into their children’s habits through cognitive socialization, behavioral modeling, and institutional resources [43].

The study also found significant physical activity risks among Crisis and Adjustment Type students. This finding is consistent with international evidence-based consensus: regular moderate-to-high-intensity physical activity is a key behavior target for improving chronic disease risks and alleviating sedentary sub-health conditions [44–46]. Crisis-type individuals often fall into a “low activity—low mood“ [47] feedback loop, reducing the executive control function of the prefrontal cortex and weakening decision-making abilities for physical activity. Their health stress can induce low mood and reduced self-efficacy [48], leading to their tendency to choose low-intensity activities (such as walking or light stretching), making it difficult to break through the threshold for upgrading their behavior [49]. Additionally, long-term observations have shown that most students, due to academic pressure, time management issues, physical education credit requirements, peer encouragement, or family support, coupled with a lack of proactive health management knowledge [50] and scientific exercise guidance [51], find it difficult to develop regular habits of moderate-to-high-intensity exercise.

## Conclusions

This study revealed a three-dimensional heterogeneous structure of college students’ healthy lifestyles through Latent Profile Analysis (LPA), with the “Sub-health Adjustment Type” serving as a key transitional state, alongside the “Health Crisis Type,” forming a high-risk dual core for insufficient physical activity. Based on the Social Ecological Model and Health Capital Theory, we suggest the following: Universities should implement brief, high-intensity training for engineering students, integrate arts therapy (such as dance-mindfulness fusion) for liberal arts students, combine clinical practice with self-care behaviors for medical students, and provide health resource compensation packages for students from rural areas. Urban students should focus on empowering health cultural capital. The interventions should primarily focus on nutrition science, exercise efficacy, psychological resilience, and stress management to build practical pathways [52], ultimately enhancing college students’ health literacy and contributing to the achievement of national health goals.

### Limitations and future directions

Although this study explored the latent categories of college students’ healthy lifestyles and their association with physical activity in Zhengzhou, enriching both theoretical perspectives and practical validation in health behavior research, it still has several limitations: (1) The study sample was restricted to students from two representative universities in Henan Province, so the results may not be directly applicable to other universities in the province; (2) Some retrospective survey questions may have led to information bias in the final results; (3) Although the IPAQ is a commonly used tool for measuring physical activity in specific settings, it tends to overestimate physical activity levels compared to objective measurement tools, including accelerometers [53, 54].

Given these limitations, the following future directions are proposed: (1) Cross-sectional designs, due to the presence of confounding factors, make it difficult to infer the association between physical activity and healthy lifestyles. Future research could consider higher evidence-level studies, such as longitudinal and randomized controlled trials, to improve the strength of the evidence; (2) Future studies could consider using accelerometers or other measurement tools to more accurately report the physical activity levels of the participants; (3) Large-scale data surveys and follow-up studies in different regions could provide richer information on physical activity and healthy lifestyles, and more precisely reveal the long-term dynamic trajectories of the association between different lifestyle categories and physical activity.

## Data Availability

The datasets generated during the current study are available from the corresponding author upon reasonable request.

## Abbreviations

WHO: World Health Organization
CSHLAS: College Students' Healthy Lifestyle Assessment Scale
IPAQ: International Physical Activity Questionnaire
BLRT: Bootstrapped Likelihood Ratio Test
LPA: Latent Profile Analysis
AIC: Akaike Information Criterion
aBIC: Adjusted Bayesian Information Criterion
BIC: Bayesian Information Criterion
NCDs: Non-Communicable Diseases
R3STEP: A robust three-step method
